# Overview of *Candida parapsilosis* candidemia in pediatric patients with hematologic and solid organ malignancies

**DOI:** 10.22034/cmm.2024.345299.1579

**Published:** 2024-12-31

**Authors:** Fatma Tuğba Çetin, Ümmühan Çay, Asena Ünal, Özlem Özgür Gündeşlioğlu, Derya Alabaz, Filiz Kibar, Nazlı Totik, Meriç Esen Şimşek Mullaoğlu, Gülay Sezgin, Serhan Küpeli

**Affiliations:** 1 Department of Pediatric Infection, Faculty of Medicine, Cukurova University, Adana, Turkey; 2 Department of Microbiology, Faculty of Medicine, Cukurova University, Adana, Turkey; 3 Department of Biostatistics, Faculty of Medicine, Cukurova University, Adana, Turkey; 4 Department of Pediatric Hematology, Faculty of Medicine, Cukurova University, Adana, Turkey; 5 Department of Pediatric Oncology, Faculty of Medicine, Cukurova University, Adana, Turkey

**Keywords:** Candidaemia, *Candida parapsilosis*, Catheter, Cancer, Pediatric patients

## Abstract

**Background and Purpose:**

Today, with the development of critical patient care and the increase in intravascular invasive methods, the survival rate of patients diagnosed with hematological and solid organ malignancies is increasing, and unfortunately, the incidence of *Candida parapsilosis* candidemia is also increasing due to multiple risk factors. In this study, we aimed to determine the clinical-demographic characteristics of *C. parapsilosis* candidemia and the antifungal susceptibility profile of *C. parapsilosis* in pediatric patients with hematological and solid organ malignancies.

**Materials and Methods:**

The present study included pediatric patients with hematologic and solid organ malignancies presenting with signs and symptoms consistent with candidemia, in whom *C. parapsilosis* was isolated from blood and catheter cultures between January 2010 and August 2023.

**Results:**

Thirty (65.2%) of the patients had hematologic and 16 (34.8%) had solid organ malignancies. In all patients, 23 (50%) had non-catheter-related candidemia and 23 (50%) had catheter-related candidemia. At least one of the risk factors examined was detected in these patients. Catheter-related candidemia was found to be more common in patients diagnosed with hematologic malignancy. The difference was found to be statistically significant (p= 0.030). Drug resistance rates of *C. parapsilosis* were 6.5% for amphotericin B, 6.5% for fluconazole, 2.2% for voriconazole and 2.2% for micafungin. No patient with caspofungin resistance was detected. The mean treatment duration of the patients was 21 days (min 3-max 103) and it was observed that amphotericin B and caspofungin were used most frequently in the treatment regimen. The mortality rate of patients with candidemia was 6.5%.

**Conclusion:**

Our study showed that patients with hematologic malignancies exhibited a higher susceptibility to catheter-related *C. parapsilosis* candidemia compared to patients with solid organ tumors. Caspofungin resistance was not detected in our study, and we believe that each center should know its own antifungal drug sensitivity, determine the treatment regimen accordingly, and that catheters should be removed rapidly in patients with catheter-related *C. parapsilosis* candidemia in malignant patients.

## Introduction

*Candida parapsilosis* is currently a significant cause of mortality and morbidity [ 1 ]. Although *Candida albicans* was previously the most prevalent species in invasive candidemia, there has been a notable surge in the incidence of *C. parapsilosis* in recent years [ 2 - 4 ]. *C. parapsilosis* represents a significant risk factor for candidemia in patients with biofilm formation and horizontal transmission from hospital personnel. It is often observed in patients with malignancy, immunocompromised status, and prolonged intensive care unit hospitalization [ 5 , 6 ]. The presence of a central venous catheter (CVC), total parenteral nutrition, prolonged hospitalization in the intensive care unit, broad-spectrum antibiotic use, and steroid use are the primary risk factors for *C. parapsilosis* candidemia [ 7 , 8 ]. In the current era, with the advent of advanced critical care and a rise in intravenous intervention, the prognosis for patients with hematologic and solid organ malignancies has improved. However, the incidence of *C. parapsilosis* candidemia has also increased. Echinocandins and amphotericin B are the preferred initial agents for the treatment of immunocompromised malignancy patients. Concurrently, the removal of the CVC is strongly advised in cases of catheter-related candidemia in accordance with the international consensus guidelines [ 9 , 10 ].

The objective of this study was to determine the clinical and demographic characteristics of *C. parapsilosis* candidemia and the antifungal susceptibility profile of *C. parapsilosis* in pediatric patients with hematologic and solid organ malignancies. 

## Materials and Methods

This study was conducted in accordance with the Declaration of Helsinki. Approval was obtained from Çukurova University Non-Interventional Clinical Research Ethics Committee University (6.9.2024).

This is a single-center, retrospective study conducted in a tertiary care hospital. The study period covered the period between January 2010 and August 2023. The study included pediatric patients with hematologic and solid organ malignancies presenting with signs and symptoms compatible with candidemia, in whom *C. parapsilosis* was isolated from blood and catheter cultures. Patients older than 18 years of age and those with incomplete data were excluded from the study.

Catheter-related candidemia was defined as candidemia diagnosed by at least one positive peripheral blood culture and accompanied by clinical signs of infection (fever, chills, and/or hypotension) in a patient with an intravenous catheter, and the absence of any other source of infection other than the catheter. In order to diagnose catheter-related candidemia, at least one of the following criteria had to be met: 1. Isolation of the same microorganism (same species and same antibiotic susceptibility pattern) in semiquantitative (>15 CFU/catheter segment) or quantitative culture (>10^3^ CFU/catheter segment) from peripheral blood and catheter. 2. A growth ratio of at least 5/1 in simultaneously obtained CVC quantitative blood culture/peripheral blood culture. Candidemia cases that did not meet the criteria mentioned above were classified as non-catheter-related candidemia [ 11 , 12 ]. In patients with a history of candidemia, the growth of *Candida* 30 days after the initial negative culture was considered to represent a new episode of candidemia. The culture and antibiogram data pertaining to candidemia were retrospectively obtained from the microbiology laboratory records. The demographic and clinical characteristics, underlying disease, risk factors, antifungal susceptibility test results, treatment, and prognosis of infections caused by *C. parapsilosis* were obtained from the patient files.

Time to *C. parapsilosis* growth in blood culture was recorded. Death within 30 days of *C. parapsilosis* growth was divided into overall death and death due to candidemia. Patients underwent abdominal ultrasonography, ophthalmologic examination, organ scanning with echocardiography, and abdominal tomography if necessary.

Clinical samples submitted to the microbiology laboratory with the requisite blood culture bottles were incubated in the BACTEC-FX-40 automatic blood culture device (Becton Dickinson, USA) for a period of seven days, with the results monitored at regular intervals. Growth signals were observed on smear preparations taken from the bottles, and gram staining was subsequently performed. Subsequently, the samples were subjected to further analysis via the use of sheep blood agar, MacConkey agar, and Sabouraud dextrose agar. Colony morphology was evaluated after 18 to 24 hours of incubation at 37°C. Isolates exhibiting yeast morphology were identified to the species level using identification cards (YST) with the VITEK 2 Compact® (bioMérieux, France) system. Due to the limitations of the laboratory conditions, it was not possible to identify any subspecies within the *C. parapsilosis* complex. Additionally, the susceptibility of the isolates to amphotericin B, fluconazole, caspofungin, micafungin, and voriconazole was determined using antifungal susceptibility cards (AST-YST01). The minimum inhibitory concentration (MIC) limit values were determined through the process of antibiotic susceptibility testing. The MIC limit value for *C. parapsilosis* was evaluated in accordance with the CLSI M60 2017 guideline [ 13 ]. A minimum inhibitory concentration (MIC) of ≥8 mg/L for fluconazole, ≥8 mg/L for caspofungin, ≥8 mg/L for micafungin, and ≥1 mg/L for voriconazole was deemed indicative of resistance [ 14 ].

The numerical data were summarized as mean± standard deviation and quartile range, while the categorical data were summarized as number (percentage). A chi-square test was employed to ascertain whether there were any significant differences between the categorical measurements. The IBM SPSS Statistics Version 20.0 (Armonk, NY: IBM Corp.) software package was employed for the analysis of the statistical data. The level of statistical significance was set at 0.05 [ 15 ]. The study was approved by the University Faculty of Medicine Hospital Clinical Research Ethics Committee (6.9.2024).

## Results

A total of 208 patients with *C. parapsilosis* candidemia were identified at Çukurova University Balcalı Hospital, Adana, Turkey, between January 1, 2010, and September 1, 2024. Of the patients mentioned above, 46 (22.1%) individuals with hematologic and solid organ malignancies were included in the study. Patients whose data could not be accessed were excluded from the study.

**Figure 1:**
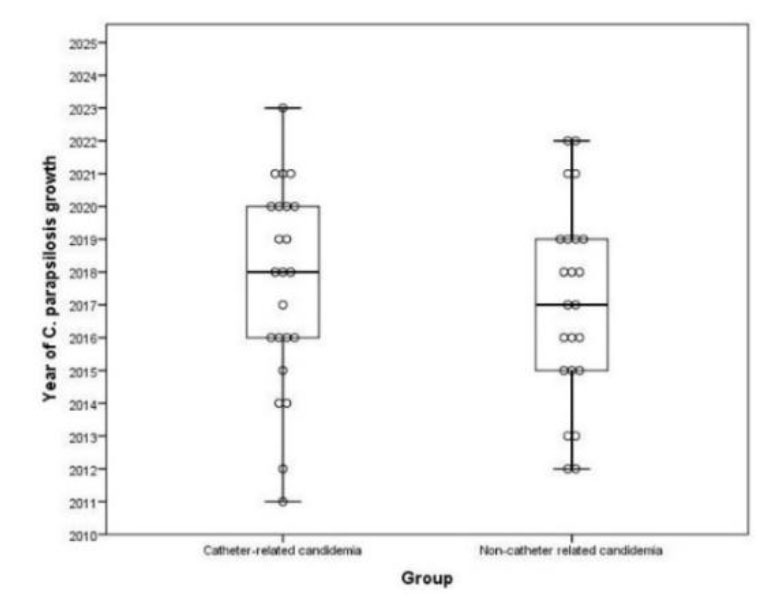
*Candida parapsilosis* candidemia distribution according to years

Thirty-six (78.3%) of the patients included in the study were male. The mean age of the patients was 87.36 months (min 4 months- max 204 months). When the patients were categorized according to age, one patient was ˃1 month- 12 months old, 15 patients were ˃1- 5 years old, and 30 patients were ˃5- 18 years old. The distribution of *C. parapsilosis* candidemia varied according to age, as shown in Figure 1. Thirty (65.2%) of the patients had hematologic malignancies, and 16 (34.8%) had solid organ malignancies. Of the patients, 42 (91.3%) had fever, 12 (26.1%) had respiratory distress, 9 (19.6%) had abdominal pain, 7 (15.2%) had vomiting, 5 (10.9%) had hypotension, 4 (8.7%) had diarrhea, 3 (6.5%) had cough, 2 (4.3%) had seizures, and 1 (2.2%) had impaired consciousness. Among all patients, 23 (50%) patients had non-catheter-related candidemia, and 23 (50%) patients had catheter-related candidemia. Of the 23 patients with catheter-related candidemia, 18 (39.1%) had port growth, three (6.5%) had CVC growth, and two (4.4%) had *C. parapsilosis* catheter tip growth. All patients with candidemia had at least one of the risk factors. Three (6.5%) patients had two risk factors, and 43 (93.5%) patients had three or more risk factors. Eight patients (17.4%) had previously received antifungal prophylaxis. Clinical and demographic characteristics, malignancy type, and candidemia risk factors of the patients are shown in Table 1.

**Table 1:** Patients' demographic and clinical characteristics

Characteristics		Number of patients n (%)
**Age (month)**	0-1 month	0 (0.0)
	1-12 months	1 (2.2)
	12-60 months	5 (32.6)
	>60 months	30 (65.2)
**Gender**	Female	10 (21.7)
	Male	36 (78.3)
**Source of candidiasis**	Catheter-related candidemia	23 (50.0)
	Port	18 (39.1)
	Central venous catheter	3 (6.5)
	Catheter tip	2 (4.4)
**Underlying malignancy**	Acute lymphoblastic leukemia	18 (39.1)
	Acute myeloblastic leukemia	12 (26.1)
	Neuroblastoma	3 (6.5)
	Lymphoma	2 (4.3)
	Brain tumor	1 (2.2)
	Retinoblastoma	1 (2.2)
	Other solid organ tumors	9 (19.6)
**Risk factor**	Antibiotic use	46 (100.0)
	Neutropenia	40 (86.9)
	Mucositis	27 (58.7)
	Taking stomach protectors	16 (34.8)
	Hypoalbuminemia	10 (21.7)
	Steroid use	9 (19.6)
	Mechanical ventilation	5 (10.9)
	Urinary catheter	5 (10.9)
	Nasogastric catheter	4 (8.7)
	Total parenteral nutrition	3 (6.5)
	History of intensive care hospitalization	2 (4.4)
**Mortality**	Overall mortality	13 (28.3)
	Candidaemia-related mortality	3 (6.5)

Catheter-related candidemia was found in 19 (63.3%) patients with hematologic malignancy and in 4 (25%) patients with solid organ tumors. Catheter-related candidemia was found to be more common in patients with hematologic malignancy. The difference was statistically significant (p= 0.030). It is shown in Table 2.

**Table 2:** Catheter-related candidemia distribution for hematological malignancy and solid organ tumor

	Catheter-related candidemia (n=23)	Non-catheter related candidemia (n=23)	p
**Hematological malignancy**	19(63.3)	11(36.7)	**0.030**
**Solid organ tumor**	4(25.0)	12(75.0)	

Data were summarized as numbers (percentages).

In three patients, there was growth accompanying the growth of *C. parapsilosis*. These were Serratia marcescens in one patient, *Candida pellucida* in one patient, and *Staphylococcus hominis* in one patient. It was noteworthy that all three patients had catheter-related candidemia. *Candida* retinitis and cardiac involvement were not detected in the patients, and three (6.5%) patients had abdominal abscess due to candidemia. It was noteworthy that the primary disease of all patients with abdominal abscess due to candidemia was hematologic malignancy and also catheter-related candidemia.

There was no statistically significant difference between catheter-related and non-catheter-related candidemia in terms of age and gender (p= 0.420, 0.722, respectively). The mean age was 6.75± 3.75 years in catheter-related candidemia and 7.82± 4.84 years in non-catheter-related candidemia. Both catheter-related and non-catheter-related candidemia were more common in men. There was no statistically significant difference between these two groups in terms of patient complaints of fever, hypotension, cough, respiratory distress, and abdominal pain (p= 0.608, 0.346, > 0.999, 0.091, > 0.999, respectively). 

**Figure 2:**
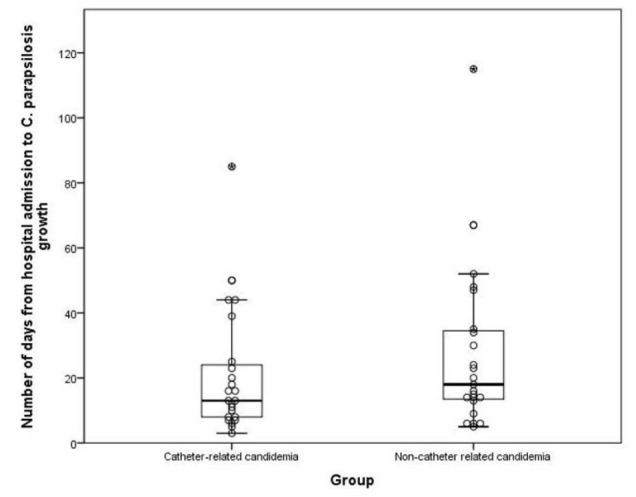
*Candida parapsilosis* reproduction time

The median day of *C. parapsilosis* growth was the 13^th^ day of hospitalization (min 3-max 85) in catheter-related candidemia and the 18th day of hospitalization (min 3- max 115) in non-catheter-related candidemia. The day of *C. parapsilosis* growth was similar between the two groups (p= 0.272) (Figure 2). The duration of culture negativity was also similar between the two groups (p= 0.240).

*C. parapsilosis* antifungal resistance was found to be 6.5% for amphotericin B, 6.5% for fluconazole, 2.2% for voriconazole and 2.2% for micafungin. No patient with caspofungin resistance was identified. *C. parapsilosis* drug resistance did not differ between malignancy types and between catheter-related and non-catheter-related candidemia in patients with malignancy (amphotericin B and fluconazole p= 0.608, voriconazole p= 0.335, micafungin p= 0.543). There were no patients with resistance to both amphotericin B and fluconazole. All three patients with abdominal abscess due to *C. parapsilosis* had no antifungal drug resistance. In addition, fluconazole resistance was not detected in patients receiving fluconazole prophylaxis. The susceptibility distribution of *C. parapsilosis* according to antifungal agents is shown in Table 3.

**Table 3:** *Candida parapsilosis* susceptibility distribution based on antifungal agents

Antifungal Medication and Sensitivity		Hematologic malignancy (n: 30)		Solid organ tumor (n: 16)		N:46
		Catheter-Related Candida (n:19)	Non-catheter-related Candidemia (n:11)	Catheter-related Candida (n: 4)	Non-Catheter-related candidemia (n: 12)	
AMB	Sensitive	18 (94.7)	9 (81.8)	4 (100.0)	11 (91.7)	42 (91.3)
	Medium sensitive	0 (0.0)	0 (0.0)	0 (0.0)	0 (0.0)	0 (0.0)
	Resistant	1 (5.3)	1 (9.1)	0 (0.0)	1 (8.3)	3 (6.5)
	Unspecified	0 (0.0)	1 (9.1)	0 (0.0)	0 (0.0)	1 (2.2)
FLC	Sensitive	19 (100.0)	8 (72.7)	3 (75.0)	12 (100.0)	42 (91.3)
	Medium sensitive	0 (0.0)	0 (0.0)	0 (0.0)	0 (0.0)	0 (0.0)
	Resistant	0 (0.0)	2 (18.2)	1 (25.0)	0 (0.0)	3 (6.5)
	Unspecified	0 (0.0)	1 (9.1)	0 (0.0)	0 (0.0)	1 (2.2)
CAS	Sensitive	15 (78.9)	9 (81.8)	3 (75.0)	11 (91.7)	38 (82.6)
	Medium sensitive	0 (0.0)	0 (0.0)	0 (0.0)	0 (0.0)	0 (0.0)
	Resistant	0 (0.0)	0 (0.0)	0 (0.0)	0 (0.0)	0 (0.0)
	Unspecified	4 (21.1)	2 (18.2)	1 (25.0)	1 (8.3)	8 (17.4)
MFG	Sensitive	14 (73.7)	7 (63.6)	3 (75.0)	8 (66.7)	32 (69.5)
	Medium sensitive	2 (10.5)	2 (18.2)	1 (25.0)	0 (0.0)	5 (10.9)
	Resistant	0 (0.0)	0 (0.0)	0 (0.0)	1 (8.3)	1 (2.2)
	Unspecified	3 (15.8)	2 (18.2)	0 (0.0)	3 (25.0)	8 (17.4)
VRC	Sensitive	19 (100.0)	8 (72.7)	4 (100.0)	12 (100.0)	43 (93.4)
	Medium sensitive	0 (0.0)	1 (9.1)	0 (0.0)	0 (0.0)	1 (2.2)
	Resistant	0 (0.0)	1 (9.1)	0 (0.0)	0 (0.0)	1 (2.2)
	Unspecified	0 (0.0)	1 (9.1)	0 (0.0)	0 (0.0)	1 (2.2

AMB; Amphotericin B, FLC; Fluconazole, CAS; Caspofungin, MFG; Micafungin, VRC; Voriconazole.

The overall mortality rate was 28.3%, and candidemia mortality rate was 6.5%. While there was no statistically significant difference in the overall mortality rate between patients with hematologic and solid organ malignancies (p= 0.493), 10 (76.9%) of the 13 patients who died from all causes were hematologic, and 3 (23.1%) were solid organ tumors. Of the three patients who died from candidemia, two were diagnosed with acute lymphoblastic leukemia and catheter-related candidemia. 

One died from Burkitt lymphoma and non-catheter-related candidemia. No drug resistance was detected in the antifungal susceptibility tests of these three patients. In one of the patients who died, *C. parapsilosis* growth was accompanied by *C. pellucida*. The three patients who died of candidemia had at least four candidemia risk factors. 

**Figure 3:**
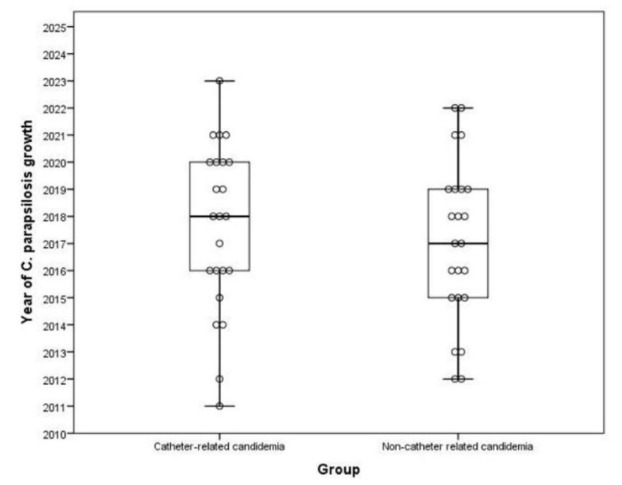
*Candida parapsilosis* candidemia distribution according to years

The mean duration of treatment was 21 days (min 3- max 103). The patient treated for three days died of candidemia on the third day of antifungal treatment. It was found that 35 (76.1%) of the patients received monotherapy (amphotericin B and caspofungin were the most common, respectively), and 11 (23.1%) received combined therapy. The patients' treatment regimens are shown in Figure 3. Of the 23 patients with catheter-related candidemia, seven (30.4%) received both lock therapy and systemic therapy because the catheter could not be removed immediately. One patient's port could not be removed because his family did not allow the port to be removed, while other patients with catheter-related candidemia had their catheters removed. The patient whose port could not be removed was treated with a lock, and the patient was discharged with oral fluconazole without port removal at the family's request. It was noted that the patient had been alive for seven years.

## Discussion

Recently, the species distribution of candidemia has shifted toward non-*albicans Candida*, especially *C. parapsilosis* species. In particular, hematologic and solid organ malignancies are defined as the main comorbidity of patients with candidemia [ 16 ]. In some Western countries, such as the USA, Australia, and Portugal, *C. parapsilosis* has been reported to be the most common cause of candidemia in patients with malignancies [ 17 - 19 ]. Most patients with malignancies are neutropenic. This is one of the major factors predisposing to invasive candidemia [ 20 ]. In our study, 86.9% of patients diagnosed with malignancy were neutropenic. In addition, mortality and morbidity in patients with malignancy are even more important with regard to *C. parapsilosis* due to its high reproductive rate and adhesion to intravenous devices and prosthetic materials [ 21 ]. In a multicenter study conducted in Greece, it was found that the most common type of candidemia in patients with hematologic and oncologic malignancies was *C. parapsilosis*, 73.7% of patients with candidemia had CVCs, and 56.3% had neutropenia [ 22 ]. In a study on candidemia in children with cancer by Barrientos et al. [ 23 ], all patients were found to be neutropenic. 

In our study, both catheter-related and non-catheter-related *C. parapsilosis* candidemia were found to be more common in male patients. Dewan et al. [ 24 ] reported that candidemia was more common in males in adult malignancy study patients. In our study, it was found that hematologic malignancy was the most common malignancy in catheter-related candidemia, and acute lymphoblastic leukemia was the most common malignancy in this group. Although this shows the importance of catheter-related candidemia in patients with hematologic malignancy, prospective studies with a larger number of patients are needed.

One study showed that the overall mortality rate in cases with candidemia in hematologic malignancy was 40%- 45% [ 25 ]. In our study, the overall mortality rate of 30 patients with hematologic malignancy was 33.3%, and the mortality rate due to *C. parapsilosis* was 6.7%. In a surveillance study conducted in Spain, the overall mortality rate of hematologic-oncologic patients was 30.9%, which was similar to ours, and the mortality rate of candidemia was 11.4%, which was higher than our study [ 26 ]. In a study conducted in Turkey, *C. parapsilosis* was found to be the most common type of catheter-related candidemia in pediatric cancer patients, and the mortality rate of candidemia was 4.44% [ 27 ].

In our study, only one of the patients with catheter-related candidemia could not have their port removed. The catheters of the other patients were removed. Lock therapy was also given in addition to systemic therapy in the six patients whose catheters were not removed immediately. Removal of the CVC and port is recommended in catheter-related candidemia. In pediatric patients, if the catheter cannot be removed immediately, intracatheter lock therapy may be beneficial in addition to systemic antibiotics depending on the etiology [ 28 ]. For *C. parapsilosis*-associated candidemia, it is clear that catheter removal is necessary, considering the adhesion of *C. parapsilosis* to invasive devices [ 26 ].

In cancer patients with pediatric candidemia, evaluation of organ involvement such as liver, spleen, heart, and eye is essential regardless of the clinical condition of the patient. In our study, hepatic candidiasis due to *C. parapsilosis* was observed in 6.5% of patients, while heart and eye involvement was not observed. In the candidemia study conducted by 29.Duzgol et al. [ 29 ] in pediatric hematologic patients, liver candidiasis was observed in 3.8% and *Candida* heart involvement in 3.8%.

Studies have reported different results regarding antifungal drug resistance. Wang et al. [ 30 ] found that the most common strain in acute leukemia patients was *C. parapsilosis*, with a resistance rate of 8.6% to fluconazole, 4.3% to amphotericin B, and no resistance to caspofungin. In our study, the resistance rates of fluconazole and amphotericin B in acute leukemia patients were 6.4%, and no resistance to caspofungin was observed. Another study investigating *C. parapsilosis* resistance in adult patients with cancer found that all patients were susceptible to fluconazole and voriconazole. In addition, the mortality rate of *C. parapsilosis* in this study was 17.5%, which was slightly higher than the rate in our study [ 31 ]. 

Two of the three patients with amphotericin B resistance received fluconazole and one received caspofungin. Of the three patients with fluconazole resistance, 1 received micafungin, 1 received caspofungin, and 1 received amphotericin B. None of the patients who died of candidemia were found to be resistant to the drug. The presence of multiple risk factors for candidemia in these patients who died despite receiving appropriate treatment significantly increased mortality. In addition, it is predicted that the response to treatment is affected by factors such as the presence of a patient's catheter, its removal, and the timing of its removal [ 29 , 32 , 33 ]. For these reasons, it is very important for these patients to receive oral care, catheter care, and neutrophil support, and to avoid unnecessary urine and nasogastric tube use.

## Conclusion

The emergence of *C. parapsilosis* as the leading non-albicans species poses a great threat to patients with malignancy. Although our study has limitations in microbiological typing of the *C. parapsilosis* complex and non-classical antifungal drug resistance, it has shown that patients with hematological malignancies are at higher risk of developing catheter-associated *C. parapsilosis* candidemia. We believe that the absence of caspofungin drug resistance in *C. parapsilosis* in malignancy patients in our center will guide our own treatment regimen. We also want to emphasize that in patients whose catheters cannot be removed rapidly, the catheter should be removed as soon as possible with lock therapy.
